# Effects of creatine monohydrate timing on resistance training adaptations and body composition after 8 weeks in male and female collegiate athletes

**DOI:** 10.3389/fspor.2022.1033842

**Published:** 2022-11-16

**Authors:** Nicholas E. Dinan, Anthony M. Hagele, Andrew R. Jagim, Michael G. Miller, Chad M. Kerksick

**Affiliations:** ^1^College of Health Professions, Midland University, Fremont, NE, United States; ^2^Graduate Program in Health Science, Rocky Mountain University of Health Professions, Provo, UT, United States; ^3^Exercise and Performance Nutrition Laboratory, College of Science, Technology, and Health, Lindenwood University, St. Charles, MO, United States; ^4^Sports Medicine, Mayo Clinic Health System, La Crosse, WI, United States; ^5^Department of Human Performance and Health Education, Western Michigan University, Kalamazoo, MI, United States

**Keywords:** supplementation, pre-workout, post-workout, peri-workout, strength, endurance, body composition, fat-free mass

## Abstract

**Background:**

Limited research is available on the potential impact of creatine monohydrate administration before or after workouts among athletes. This study aimed to investigate the effects of pre- vs. post-exercise creatine monohydrate supplementation on resistance training adaptations and body composition.

**Methods:**

In a randomized, double-blind, placebo-controlled, parallel design, 34 healthy resistance-trained male and female athletes were randomly assigned and matched according to fat free mass to consume a placebo, or 5-g dose of creatine monohydrate within 1 h before training, or within 1 h after training for 8 weeks, while completing a weekly resistance training program. Participants co-ingested 25-gram doses of both whey protein isolate and maltodextrin along with each assigned supplement dose. Body composition, muscular strength, and endurance, along with isometric mid-thigh pull were assessed before and after the 8-week supplementation period. A 3 × 2 mixed factorial (group x time) ANOVA with repeated measures on time were used to evaluate differences.

**Results:**

All groups experienced similar and statistically significant increases in fat free mass (+1.34 ± 3.48 kg, *p* = 0.04), upper (+2.21 ± 5.69 kg, *p* = 0.04) and lower body strength (+7.32 ± 10.01 kg, *p* < 0.001), and decreases in body mass (−1.09 ± 2.71 kg, *p* = 0.03), fat mass (−2.64 ± 4.16 kg, *p* = 0.001), and percent body fat (−2.85 ± 4.39 kg, *p* < 0.001).

**Conclusions:**

The timing of creatine monohydrate did not exert any additional influence over the measured outcomes.

## Introduction

Ergogenic aids are commonly used to improve performance or augment exercise training adaptations (1, 2). Creatine monohydrate (1, 3–6) supplementation is well-established for its ability to increase intramuscular creatine concentration and its subsequent ergogenic potential as seen by commonly reported increases in fat-free mass accretion, augmented muscle morphology changes, and improvements in muscular strength, endurance, and power (3, 7, 8). Altering the timing of when nutrients are delivered has been established as a potential strategy to augment recovery from stressful exercise while also impacting adaptations to regular exercise training (9). While the majority of the research regarding the effects of nutrient timing strategies has centered upon the delivery of macronutrients (9), interest exists to better understand how the timing of other micronutrients or single ingredients, such as creatine monohydrate, may affect recovery and exercise training adaptations (10). For example, early work (11) demonstrated that ingesting a combination of carbohydrate + protein + creatine (Cr) in close proximity (vs. morning and evening ingestion) to each workout over 10 weeks, elicited greater increases in intramuscular creatine, lean tissue accretion, cross-sectional area of type II muscle fibers, and exercise performance were observed. While this study highlighted the potential advantage of ingesting creatine in close proximity to a workout, additional questions remain whether or not ingesting creatine monohydrate in isolation, before or after exercise sessions, offers any advantages. In 2013, Antonio and Ciccone (12) conducted a 4-week resistance training and concluded that creatine ingestion (5 grams) post-exercise led to greater improvements in fat-free mass and strength when compared to ingesting an identical 5-gram dose before each workout. Conclusions from this study were limited, however, due to the short supplementation period, no placebo group, and the statistical approach employed by the investigators. Candow et al. later published two separate investigations in older adults spanning 12 weeks (13) and 8 months (14), respectively, which both reported no differential impact of creatine timing on resistance training adaptations. Similarly, a 2021 study by Forbes et al. (15) indicated that Cr timing did not alter improvements in muscle thickness and strength after 8 weeks of supplementation (2 days per week on training days only) while following a unilateral resistance training program. As it stands, more research is needed to better investigate the impact of creatine timing on exercise training adaptations in young, athletic populations over several weeks of exercise training and supplementation. Therefore, the aim of the current study was to evaluate the effects of 8 weeks of timed creatine supplementation on resistance training adaptations in college-aged male and female athletes. Based upon previous research which has highlighted an upregulation of anabolic signaling mechanisms (16) and hyperemia post-exercise (17), coupled with an increase an insulin sensitivity from the added carbohydrate (18), we hypothesize that post-exercise creatine consumption would lead to increased kinetics pertaining to creatine transport and result in greater improvements in strength and body composition throughout the study protocol.

## Methods

### Experimental design

In a double-blind, placebo-controlled, parallel design, study participants were randomized based upon fat-free mass into one of three groups to ingest creatine (before or after workouts) or a placebo daily throughout an 8-week supplementation period. Study participants were randomized into one of three supplement groups using block randomization, counter-balanced for sex, and matched for fat-free mass. Each group consumed their assigned dose or placebo within 1 h before exercise or within 1 h following exercise and were recommended to consume first thing in the morning and in the evening, after 5:00 p.m. hours on non-training days.

**Figure 1:**
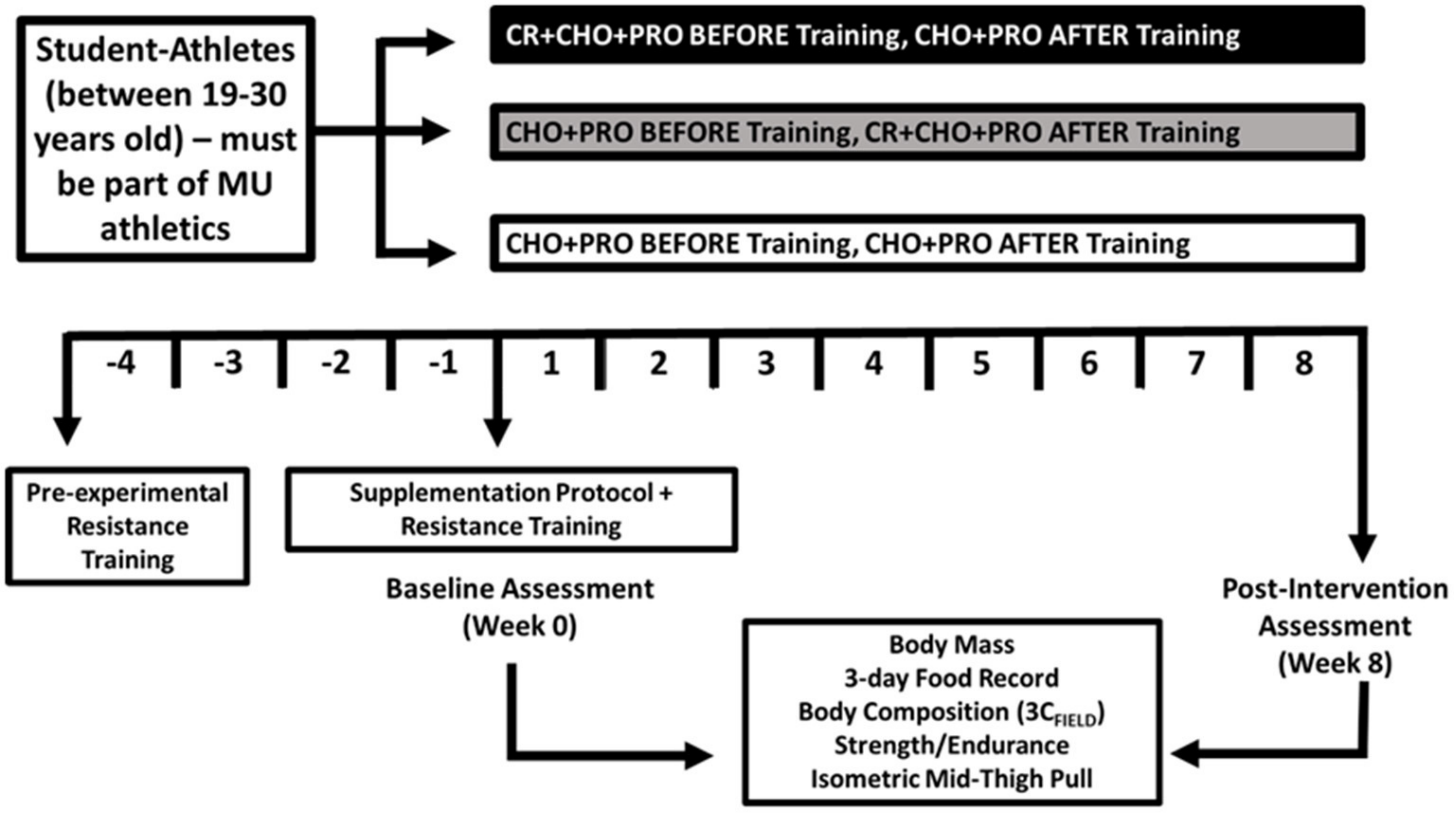
Research design overview.

All participants completed a 12-week resistance training and conditioning program. To homogenize neurological responses to resistance training, a 4-week (no supplementation) run-in period was completed, prior to participants completing baseline testing and began the supplementation protocol, while continuing to follow the resistance training program for an additional 8 weeks. Before and after the supplementation and resistance training period, body mass, body water, and body composition were assessed in addition to changes in muscular strength, muscular endurance, and lower-body power (Figure 1). This study was retroactively registered on ClinicalTrials.gov as NCT05451498.

### Study participants

Thirty-four apparently healthy male (*n* = 18) and female (*n* = 16) collegiate athletes (mean ± SD; age = 19.8 ± 1.5 years; height = 175.1 ± 9.7 cm; body mass = 82.5 ± 21.9 kg; body mass index = 26.5 ± 4.8 kg/m^2^) from women's volleyball (*n* = 3), women's soccer (*n* = 13), and football (*n* = 18) volunteered to participate in this study. To be eligible, participants were required to be current members of the university's athletic program, participating in their prescribed off-season resistance training program, between the ages of 19–30, and medically cleared to participate in their respective sport. Exclusion criteria included, any individual who is currently being treated or is diagnosed with a cardiac, respiratory, circulatory, musculoskeletal, metabolic obesity (defined as body mass index >35 kg/m^2^ and body fat >30%), immune, autoimmune, psychiatric, hematological, neurological, or endocrinological disorder or disease, anabolic steroid use, allergies to milk or soy (lecithin), and are currently pregnant or become pregnant at any point throughout the study (females only). Further, participants were required to stop taking any ergogenic agents (pre-workouts, creatine, protein, beta-alanine, etc.) 4weeks prior to and throughout the entire study protocol.

**Figure 2:**
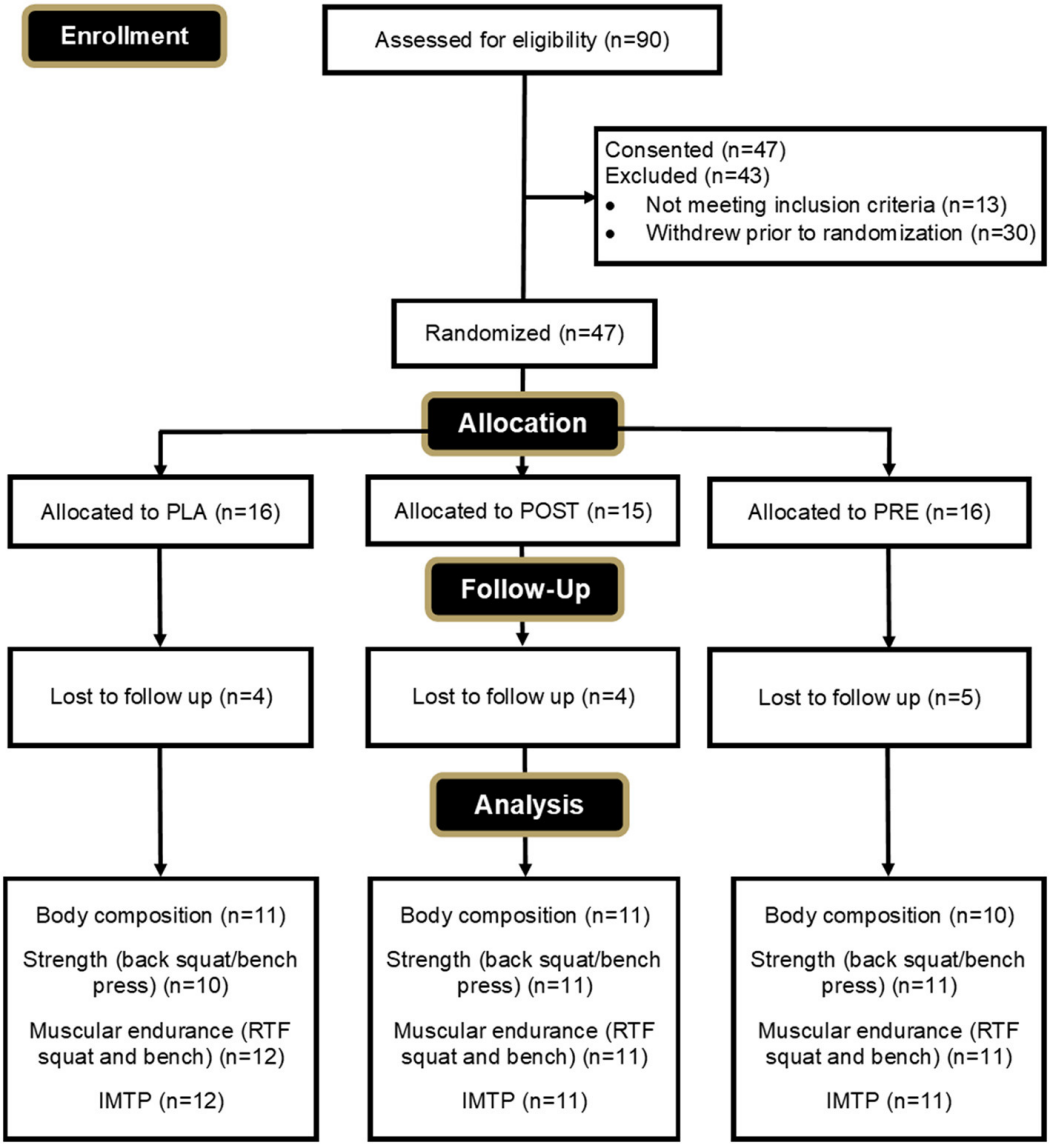
CONSORT diagram.

A CONSORT diagram is provided in Figure 2 to outline the enrollment and group allocation process. The experimental protocol was approved by the Rocky Mountain University of Health Professions Institutional Review Board on 9 February 2022, with code 2021-28. All participants were informed of their obligations and risks associated with the study protocol and provided their written consent on an IRB-approved informed consent document prior to participation.

### Anthropometric assessments

Before each visit, participants were instructed to fast overnight and abstain from exercise, caffeine, nicotine, and alcohol for at least 24 h. During the initial assessment, participant height was assessed to the nearest ± 0.5 cm using a stadiometer with their shoes removed and standing erect on flat feet. Body mass was measured prior to all study visits using a self-calibrating digital balance (Tanita BWB-627A, Tokyo, Japan) and was recorded to the nearest ± 0.1 kg.

### Body composition

A field-based three-compartment (3C_FIELD_) model was utilized to estimate body composition and determine fat mass (FM), fat-free mass (FFM), and total body water (TBW) by combining estimates of body density (BD) derived from skinfold measurements, and TBW using bioimpedance spectroscopy (BIS) (19–21). This combination of methods has been previously shown to provide more accurate body composition estimates than if used as individual techniques (20). Further, recent evidence suggests that minimal differences exist between a 3C and 4C model (19, 22).

### Total body water

To ensure adequate hydration, participants were instructed to follow a hydration protocol as hydration status was not assessed prior to testing. Total body water (TBW) was assessed using bioelectrical impedance spectroscopy (SFB7, Impedimed Corp., Carlsbad, CA) as previously described (23). Test-retest reliability using bioelectrical impedance spectroscopy is available for fat mass (CV: 5.86%, ICC: 0.98, SEM: 280.9 grams) and fat-free mass (CV: 1.72%, ICC: 0.99, SEM: 285.1 grams) in a cohort (*n* = 40) of healthy college-aged men and women (23).

### Skinfolds

Skinfold measurements were completed on the right side of the body by the same trained investigator according to the recommendations by Jackson and Pollock (24). A calibrated Lange caliper was used to take duplicate measurements at each of the seven sites (chest, mid-axilla, triceps, abdomen, suprailium, subscapular, and thigh) for both men and women and used to estimate body density using the Siri equation (25). Fat-free mass, fat mass, and body fat percentage then calculated using a 3C model (20) were recorded. Skinfold technician test-retest reliability in the current study was CV: 0.35%, ICC: 0.985 (95% CI: 0.976, 0.991), SEM: 1.37 millimeters.

### Isometric mid-thigh pull testing

A standardized warmup consisting of dynamic bodyweight movements including walking lunges, squats, and leg swings was completed prior to assessing maximal torque using an isometric mid-thigh pull (IMTP) as previously described (23). Study participants completed three, maximal-effort attempts with 60 s of rest allowed between each attempt. The highest value was recorded and used for later analysis. Isometric mid-thigh pull assessments have been shown to be strongly associated with athletic performance outcomes such as strength (26, 27), sprint performance (27, 28), and agility performance (27).

### Maximal strength and muscular endurance

Maximal strength [one-repetition maximum (1 RM)] and muscular endurance [repetitions to fatigue (RTF) at 80% 1 RM] were assessed in the following sequence: (a) back squat strength, (b) back squat endurance, (c) bench press strength, and (d) bench press endurance. Approximately 5 min of rest separated each assessment. All protocols were consistent with the National Strength and Conditioning Association (NSCA) and previously described (27, 28).

### Resistance training program

As highlighted previously, all study participants completed 12 weeks of resistance training at a frequency of 4 days per week which was designed and supervised by the university strength and conditioning coach. The first 4 weeks of resistance training (16 workouts) commenced before the initiation of the supplementation period and baseline testing to acclimate participants and homogenize training responses to the program. Following the initial 4-week resistance training block, an additional 8 weeks of resistance training was completed (~32 workouts) that coincided with the supplementation protocol. Each workout consisted of mostly multi-joint exercises with free weights that targeted all major muscle groups [i.e., rack/hang cleans, bench press (flat and incline), push press, rear deltoid rows/pull apart, pendlay rows, low rows, pulldowns, triceps extensions, bicep curls, back and belt squats, front and side planks, glute/ham raises, and box/seated jumps]. A progressive overload scheme was followed to facilitate increases in strength and lean body mass. The program was divided into three phases: phase 1 (< 60–70% 1 RM) for weeks 1–4, phase 2 (70–80% 1 RM) for weeks 4–8, and phase 3 (80–95% 1 RM) for weeks 8–12. Attendance was required daily and compliance was calculated as the percentage of completed workouts. Participants were deemed compliant if they completed at least 90% of the assigned workouts throughout the study protocol.

### Dietary protocol

Participants were requested to submit a three-day dietary intake log two times throughout the study (weeks 0 and 8). Study participants used MyFitnessPal (Under Armor, Baltimore, MD) to create individualized profiles and self-report energy and macronutrient intake. The 3-day average was computed for later analysis, however, compliance to completion of the dietary records was poor with only five of the 34 athletes completing all requested days.

### Supplementation protocol

Two doses of the assigned supplement (or placebo) were ingested each day by all study participants for 8 weeks. In a double-blind manner, the PRE group was instructed to ingest a 5-gram dose of creatine before exercise and a 5-gram dose of placebo (maltodextrin) after exercise. The POST group ingested a 5-gram dose of placebo (maltodextrin) before exercise and ingested a 5-gram dose of creatine after exercise. The PLA group ingested a 5-gram dose of placebo (maltodextrin) before exercise and a 5-gram dose of placebo (maltodextrin) after exercise. All doses were ingested 1 h before exercise and within 1 h after exercise. On non-training days, participants ingested the assigned supplement dose first thing in the morning (immediately upon waking) and their other assigned dose after 5:00 p.m. Participants consumed each assigned supplement along with a 25-gram dose of whey protein isolate and a 25-gram dose of carbohydrate powder. The carbohydrate and protein were added to aid in blinding, promote compliance, and facilitate optimal exercise training adaptations throughout the study protocol. Compliance to the supplementation regimen was not monitored as the principal investigator and the head strength and conditioning coach were able to communicate daily with participants regarding their compliance to the study protocol.

All supplements were provided to participants in powder form and were of similar texture, bitterness, appearance, and sweetness. All supplements were weighed and blinded by research personnel not involved in testing. The whey protein isolate and maltodextrin were provided by Argopur Dairy Cooperative (La Crosse, WI) and the creatine was provided by 1st Phorm, LLC (St. Louis, MO).

### Adverse event reporting

The occurrence of adverse events was collected through spontaneous reporting by the study participants or the interaction of the principal investigator with study participants. When participants reported adverse events, they reported the frequency as well as the severity (“mild”, “moderate”, “severe”) of any adverse event.

### Statistical analysis

All analyses were completed using Microsoft Excel and the Statistical Package for the Social Sciences (v23; SPSS Inc., Chicago IL). Data were considered statistically significant when the probability of a type I error was 0.05 or less. Primary endpoints for this investigation were changes in fat-free mass and back squat 1 RM. Secondary endpoints were fat mass, % fat, total body water, bench press 1 RM, bench press repetitions to fatigue, and back squat repetitions to fatigue. A 3 × 2 mixed factorial (group × time) ANOVA with repeated measures on time were used to determine any statistically significant differences for time and group main effects and group × time interaction effects. All data are presented as means ± standard deviations and the presence of any statistical outlier was evaluated by Grubbs' test and removed from analysis. Reliability statistics were determined using intraclass correlation and agreement between Pearson correlations.

## Results

### Participants

Of the 47 participants who were randomized and enrolled in the study, eight (PRE = 2, POST = 4, PLA = 2) withdrew due to personal reasons unrelated to the study, and one participant was non-compliant with the protocol (PLA = 1). An additional four participants (PRE = 2, PLA = 2) withdrew due to undesired side effects relating to weight gain that were possibly related to supplementation. In total, 34 participants completed the study and were included in the final analysis (PRE = 12; POST = 11; PLA = 11). Final compliance with recording their workout information was 90.4% for PRE-group, 94.5% for POST-group, and 93.3% for PLA-group.

### Adverse events

Throughout the study, 17 adverse events were reported (13 were abdominal symptoms, three were nausea, and one was due to periodic headaches). Of these, *n* = 9 were mild, *n* = 7 were moderate, *n* = 1 severe. The severe adverse event (POST = 1) was related to dizziness but was determined to not be related to the study. All adverse events were resolved by the end of the first 2 weeks. One participant who reported an adverse event (PLA = 1) withdrew due to reasons directly related to supplementation.

### Baseline differences

One-way ANOVA on pre-supplementation data indicated no baseline differences (*p* > 0.05) between groups for age (*p* = 0.21), height (*p* = 0.36), body mass (*p* = 0.69), body mass index (*p* = 0.76), % body fat (*p* = 0.26), fat-free mass (*p* = 0.73), back squat 1RM (*p* = 0.69), bench press 1 RM (*p* = 0.63), and IMTP peak force (*p* = 0.55).

### Dietary intake

Dietary data were collected during the first (PRE = 4; POST = 6, PLA = 4) and final (PRE = 3; POST = 2; PLA = 1) week of supplementation. Using a One-way ANOVA, no differences in dietary intake (i.e., total energy, carbohydrate, fat, and protein; Supplementary Table 1) were observed at baseline between groups for energy and macronutrient intake. Due to limited post-protocol data, paired-samples *t*-test were utilized to determine changes from baseline. No differences were observed between data points across time (*p* > 0.05). Similar outcomes were revealed when all data were represented relative to body mass (Supplementary Table 1).

### Anthropometrics and body water

Supplementary Table 2 presents body mass, total body water, intracellular water (ICW), extracellular water (ECF), and body composition changes. No significant (*p* > 0.05) group × time interaction effects were observed for body mass, body mass index, body water compartments, or body composition parameters. Main effects for time were observed for body mass (*p* = 0.03, ηp2 = 0.15), body mass index (*p* = 0.03, ηp2 = 0.15), total body water (*p* = 0.02, ηp2 = 0.18) and intracellular water (*p* = 0.006, ηp2 = 0.22).

### Body composition

**Figure 3:**
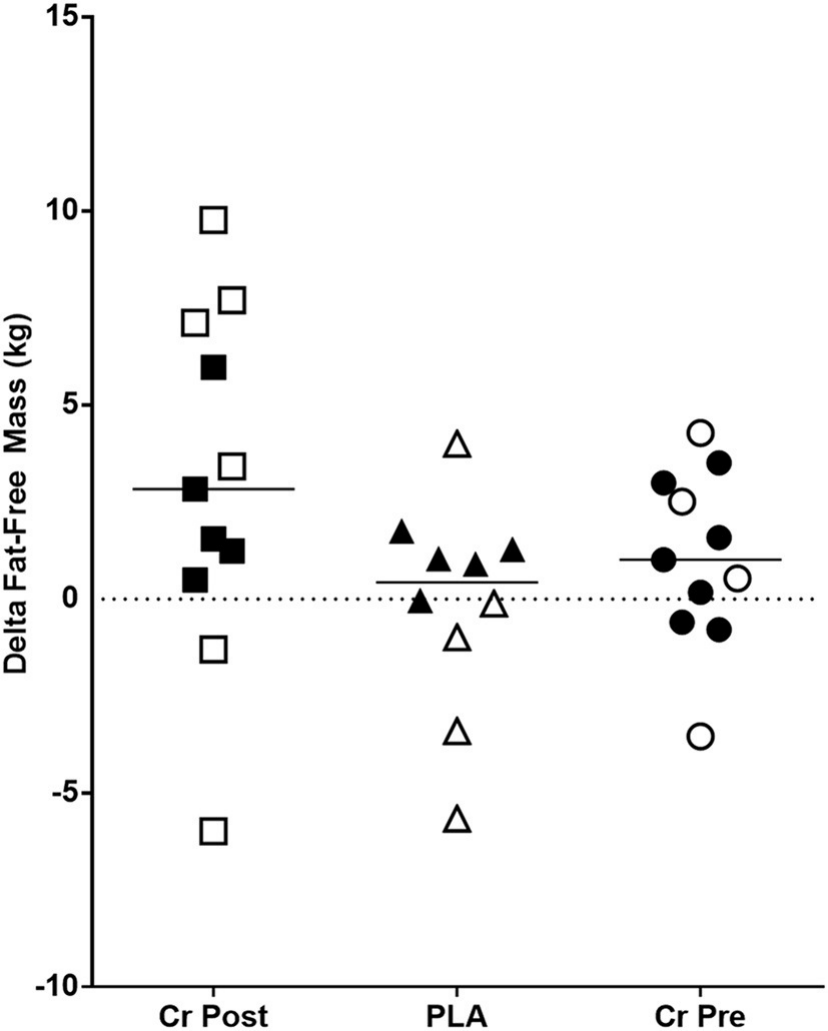
Change in fat-free mass (Week 8 - Week 0) in kilograms. Cr Post, Post-exercise creatine supplementation; Cr Pre, Pre-exercise creatine supplementation; PLA, Placebo supplementation. For each respective shape, the filled shapes (■▴•) are female participants, and the non-filled shapes (△°□) are male participants.

No significant group × time interaction effects were observed for fat-free mass (*p* = 0.11), fat mass (*p* = 0.33), and % body fat (*p* = 0.38). Main effects for time were observed for fat free mass (Figure 3) (*p* = 0.02, ηp2 = 0.17), % fat (*p* < 0.001, ηp2 = 0.35), and fat mass (*p* < 0.001, ηp2 = 0.33) (Supplementary Table 2).

### Exercise performance

#### Muscular strength and endurance

As seen in Supplementary Table 3, no significant (*p* > 0.05) group x time interaction effects were observed for any back squat or bench press performance parameters. Significant main effects for time were observed for back squat 1RM (*p* < 0.001, ηp2 = 0.35), back squat 1RM normalized to body mass (*p* < 0.001, ηp2 = 0.45), bench press 1RM (*p* = 0.04, ηp2 = 0.13), and bench press 1RM normalized to body mass (*p* < 0.001, ηp2 = 0.32), while no significant (*p* > 0.05) changes over time were observed for any other performance parameter (Supplementary Table 3). Finally, total resistance training load-volume (sets × reps × load) was calculated for the entire 8-week protocol. No significant differences between groups were found for total load-volume (PRE: 58,505 ± 35,887 kg vs. POST: 88,873 ± 58,672 kg vs. PLA: 70,408 ± 43,928 kg, *p* = 0.32, ηp2 = 0.07).

## Discussion

**Figure 4:**
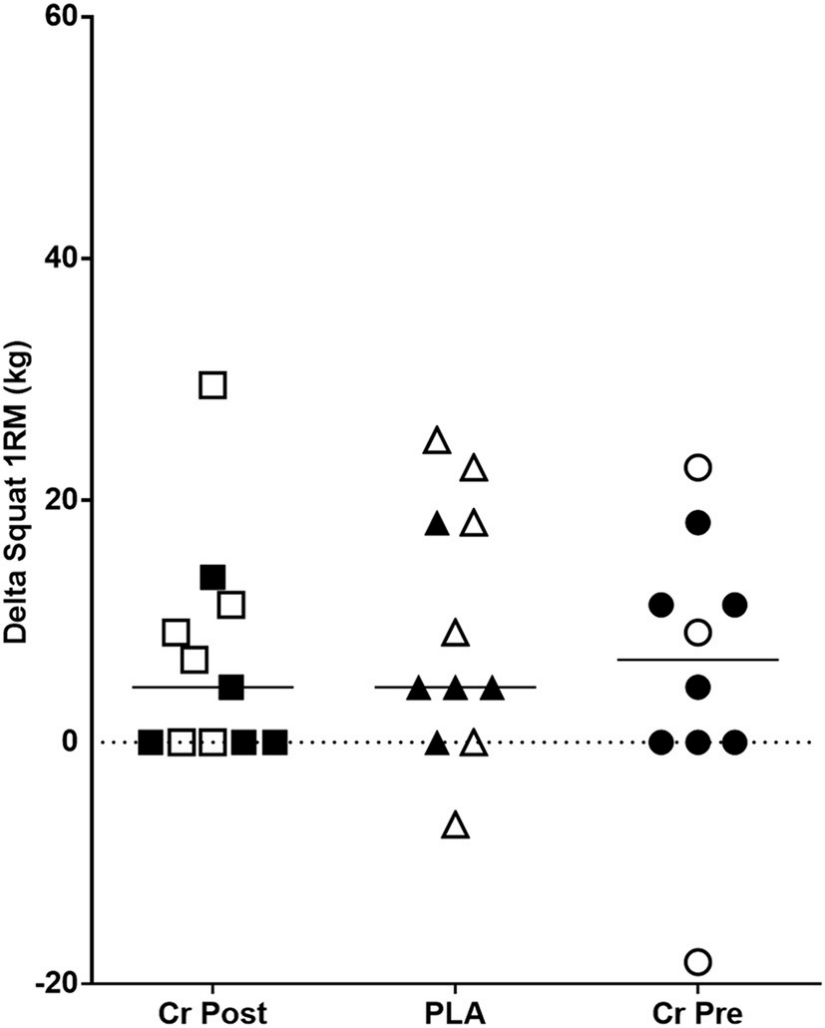
Change in squat 1RM performance (Week 8 - Week 0) in kilograms. Cr Post, Post-exercise creatine supplementation; Cr Pre, Pre-exercise creatine supplementation; PLA, Placebo supplementation. For each respective shape, the filled shapes (■▴•) are female participants, and the non-filled shapes (△°□) are male participants.

The purpose of this investigation was to examine the effects of timed creatine monohydrate supplementation (pre- vs. post-exercise) on resistance training adaptations in college-aged male and female athletes. Results from this investigation indicated that timing of creatine monohydrate exerted no further change in key outcomes such as fat-free mass (*p* = 0.11, Figure 3) and back squat 1RM (*p* = 0.87, Figure 4) in addition to other performance and body composition variables (see Supplementary Tables 2, 3). Beyond this observation, we also reported significant improvements in several body composition and performance variables, which provide sound evidence that the training program and supplementation provided throughout this study instigated changes in all study participants from baseline. Overall, the key findings from the present investigation indicate that the ingestion, nor the timing, of creatine monohydrate in combination with carbohydrate and whey protein did not exert any influence on performance or body composition outcomes in healthy, college-aged male and female athletes following 8-weeks of resistance training.

The primary rationale for this investigation was based upon the combined tenets of creatine supplementation (3, 29) and nutrient timing (9). In this respect, previous research has established the efficacy of combining creatine supplementation with resistance training (3, 7, 8, 30, 31) for its ability to augment adaptations over time, while several investigations and reviews have highlighted the potential impact of nutrient timing (9–11, 32–34). Currently, a small number of original investigations have explored the potential efficacy of timed creatine monohydrate administration, displaying mixed outcomes. In this respect, a recent review paper that discussed creatine timing (35) included six studies, with only two studies providing some level of evidence for heightened adaptations in regards to the manipulation of when creatine was ingested, in favor of post-exercise Cr supplementation (as compared to pre-exercise) (12, 14). From these studies, one should consider that the Candow study (14) was completed in elderly individuals, which limits its generalizability to younger, athletic populations and the primary population upon which the efficacy of creatine supplementation has been built. Moreover, the Antonio study (12), while completed in healthy active men, lasted 4 weeks in duration, had no placebo group, employed a liquid creatine formulation with no loading phase, did not assess body composition or lower-body performance changes, and used magnitude-based inferences; a statistical approach that has been challenged by some scientific journals and statisticians (36–38). Outside of these two investigations, the remaining studies have failed to identify any differences in resistance training adaptations between pre- vs. post-exercise consumption of creatine monohydrate. Therein, the results from the present investigation are in agreement with those studies which did not identify any added benefit of ingesting a daily dose of creatine monohydrate either before or after resistance exercise and therefore does not support our hypothesis. It is possible that the post-exercise hyperemia (17) and purported enhancement of creatine transport *via* increased insulin sensitivity (18) was not sufficient to elicit meaningful differences in measured outcomes over time; however, more work is needed to identify the specific mechanism of action.

Results from the present investigation also indicate that a daily dose of creatine monohydrate did not exert any additional benefit for improvements in body composition or performance outcomes, when compared to the placebo group; in the presence of provisional protein and carbohydrates. While somewhat unexpected due to the widespread literature supporting the benefits of creatine, the exclusion of a loading phase in our study as well as the co-ingestion of carbohydrate and protein on two occasions each day, may have limited the ability to discern differences between groups over the time frame within which our study was completed. In this respect, a loading phase is commonly employed with creatine monohydrate supplementation studies, with the goal of robustly increasing intramuscular phosphocreatine levels by 20–40% in a 5–7-day time period (39). We chose not to employ a loading phase due to the challenges associated with how to execute a loading phase, while also maintaining the blinding and timing intervention that was central to this project's aim. Previous work by Hultman et al. (39) has shown that after ~28 days of single 5-gram doses of creatine intramuscular creatine is significantly increased, which, in the present study, should have allowed for an additional 4 weeks to examine if the timing of creatine was relevant. Another key consideration was that we decided to provide two daily doses of 25 grams of maltodextrin carbohydrate and 25 grams of whey protein isolate, which provided an additional 50 grams of carbohydrate and 50 grams of whey protein isolate per day to all study participants. This decision was made for three primary reasons. First, to help ensure each participant was provided with an efficacious dose of essential amino acids and energy to promote an anabolic environment throughout the study protocol. Previous research has demonstrated that essential amino acids (EAA), in optimal dosages, maximally stimulates rates of muscle protein synthesis, particularly when ingested in close proximity to a resistance-training bout, and also that the presence of CHO may further enhance this response (8, 40, 41). Moreover, when creatine is added to whey protein, studies have indicated that a greater improvement in lean mass may occur when compared to whey protein or CHO alone (42). Thus, it is possible that any additional ergogenic potential derived from creatine administration was clouded by co-ingestion of protein and carbohydrates and the absence of a loading phase. Certainly, one could point to the findings of Cribb and Hayes (11) to refute our suggestion that added carbohydrate and protein clouded our ability to identify creatine-mediated changes, but the dosing regimen provided by Cribb and Hayes also delivered over two times the amount of creatine each day as what was delivered in the present study, which likely maximized intramuscular creatine much quicker than the dosing regimen utilized in the current study. The inclusion of a true control group in the Cribb study could have helped to further explore this possibility. The second reason for co-ingestion of carbohydrates and protein was to aid in blinding the administration of either creatine monohydrate or the placebo to our study participants. The third and final reason was to improve recruitment efforts, whereby all participants were minimally provided two daily doses of carbohydrates and protein to help maximize the potential for augmented training outcomes as no other compensation was provided.

The overall training outcomes realized by the resistance training program from the current study were largely consistent with other commonly reported training adaptations following off-season strength and conditioning programs in the literature (43–47). In this regard, main effects over time were observed, which illustrated improvements in upper- (2.8%) and lower-body strength (7.3%), as well as positive adaptations to several body composition outcomes including fat-free mass, fat mass, percent fat, and total body water. The decrease in body mass was somewhat unexpected, but the positive changes in fat-free mass, fat mass, and percent body fat do align with the observed changes in body mass.

Several strengths are evident from the present study starting with the randomized, double-blind, placebo-controlled approach, with more study participants per group than what has been previously reported in the literature (12, 13, 15). Another key strength was the 4-week period of resistance training that occurred in all supplementation groups prior to initiation of the supplementation protocol. This decision was made due to the variety of ages and genders of participants in the present study. While it is acknowledged that 4 weeks of training does not replace the neuromuscular adaptation observed with more advanced training ages, the younger training ages of some of the study participants did likely benefit from this period of resistance training. Certainly, our study was not without limitations. Most notably was our extremely poor compliance to recording of dietary intake. As mentioned previously in the paper and despite repeated reminders and efforts by the research team to complete food records, we are left with very limited data to quality dietary intake throughout the study. While we are encouraged by the significant main effects of time observed for fat-free mass accretion in all groups (Figure 3), we are not able to communicate how the quality of the diet consumed did or did not further support these observed changes. Thus, the reader is strongly encouraged to consider this when evaluating our findings and conclusions. Future research should seek ways to maximize dietary intake reporting by their study cohorts. The lack of dual x-ray absorptiometry (DEXA) to assess body composition would have provided a more robust body composition measure [i.e., four compartment (4C) model], although some have argued there is minimal difference between a 3C (used in the present study) and 4C model (19, 22). The low compliance regarding dietary intake logs was another shortcoming of the current study. Additionally, no measure of hydration status was taken, although participants were strongly encouraged to follow a hydration protocol before testing sessions. Lastly, no measures of initial creatine levels, muscle fiber morphology, blood flow kinetics, muscle cross-sectional area, myogenic transcription factors, or hormonal properties were measured in the current study as one or all of these measures would have helped to mechanistically explain some of our findings.

In conclusion, the current investigation examined the impact of 8 weeks of timed creatine monohydrate supplementation (pre-exercise vs. post-exercise) on resistance training adaptations in college-aged male and female athletes. It was revealed that the timing of creatine monohydrate in combination with carbohydrate and whey protein did not exert any differential effects for performance or body composition outcomes in healthy, college-aged men and women.

## Data availability statement

The raw data supporting the conclusions of this article will be made available by the authors, without undue reservation.

## Ethics statement

This study involving human participants was reviewed and approved by Rocky Mountain University of Health Professions Institutional Review Board. The participants provided their written informed consent to participate in this study.

## Author contributions

ND, AJ, MM, and CK designed study. ND, AH, AJ, and CK did data collection. ND and CK analyzed data and prepared initial draft. All authors approved final version.

## Funding

This research was partially supported by a research grant from Rocky Mountain University of Health Professions and internal funding provided by the Exercise and Performance Nutrition Laboratory at Lindenwood University.

## Conflict of interest

The authors declare that the research was conducted in the absence of any commercial or financial relationships that could be construed as a potential conflict of interest.

## Publisher's note

All claims expressed in this article are solely those of the authors and do not necessarily represent those of their affiliated organizations, or those of the publisher, the editors and the reviewers. Any product that may be evaluated in this article, or claim that may be made by its manufacturer, is not guaranteed or endorsed by the publisher.
