# Supplementary material for: Effects of creatine monohydrate timing on resistance training adaptations and body composition after 8 weeks in male and female collegiate athletes
Source: Front Sports Act Living. 2022 Nov 16;4:1033842. doi: 10.3389/fspor.2022.1033842 (PMC9708881; doi:10.3389/fspor.2022.1033842)
Supplement: Supplementary file 1 [file Table_1.pdf]

Supplementary Data Table 1: Dietary Variables.

Variable	Group	Baseline (Week 0)	Post-Test (Week 8)	One-Way ANOVA (Baseline only) (p)	Within Group (Paired Samples T-Test) (p)
**Energy Intake (kcal/day)**	PRE	2357 ± 888	2497 ± 1033	0.50	0.58
	POST	2090 ± 416	1852 ± 586		
	PLA	2570 ± 454	2926		
**Relative Energy Intake (kcal/kg)**	PRE	33.9 ± 9.9	37.8 ± 7.0	0.42	0.57
	POST	26.4 ± 7.7	30.6 ± 7.7		
	PLA	30.1 ± 8.2	29.6		
**Carbohydrate (g/day)**	PRE	301.8 ± 60.8	321.8 ± 135.5	0.09	0.18
	POST	216.4 ± 48.6	206.8 ± 32.8		
	PLA	286.6 ± 69.8	282.3		
**Relative Carbohydrate (g/kg/day)**	PRE	4.6 ± 1.6	4.9 ± 0.7	0.13	0.57
	POST	2.7 ± 0.9	3.4 ± 0.3		
	PLA	3.4 ± 1.3	2.9		
**Protein (g/day)**	PRE	121.9 ± 41.3	111.2 ± 38.4	0.49	0.68
	POST	130.1 ± 22.8	110.8 ± 32.7		
	PLA	147.1 ± 24.1	165.3		
**Relative Protein (g/kg)**	PRE	1.7 ± 0.3	1.7 ± 0.2	0.86	0.75
	POST	1.6 ± 0.4	1.8 ± 0.4		
	PLA	1.7 ± 0.3	1.7		
**Fat (g/day)**	PRE	71.8 ± 46.6	83.4 ± 36.7	0.36	0.20
	POST	76.0 ± 22.7	62.7 ± 36.7		
	PLA	101.5 ± 21.5	125.3		
**Relative Fat (g/kg)**	PRE	0.9 ± 0.3	1.3 ± 0.4	0.87	0.17
	POST	0.9 ± 0.3	1.0 ± 0.5		
	PLA	1.2 ± 0.3	1.3		

Baseline (Week 0), PRE, n=4; POST, n=6; PLA, n=4.

Post-Test (Week 8), PRE, n=3; POST, n=2; PLA, n=1.
