# Supplementary material for: Effects of creatine monohydrate timing on resistance training adaptations and body composition after 8 weeks in male and female collegiate athletes
Source: Front Sports Act Living. 2022 Nov 16;4:1033842. doi: 10.3389/fspor.2022.1033842 (PMC9708881; doi:10.3389/fspor.2022.1033842)
Supplement: Supplementary file 2 [file Table_2.pdf]

Supplementary Data Table 2. Body Composition Variables.

Variable	Group	Baseline (Week 0)	Post-Test (Week 8)		*p*
**Body Mass (kg)**	PRE	79.8 ± 21.9	79.3 ± 20.9†	Time	0.03
	POST	87.3 ± 26.4	85.7 ± 24.4†	Group	0.70
	PLA	80.7 ± 19.7	79.5 ± 16.4†	G x T	0.70
**Body Mass Index (kg/m^2)**	PRE	26.7 ± 5.3	26.5 ± 5.1†	Time	0.03
	POST	27.2 ± 5.6	26.8 ± 4.9†	Group	0.75
	PLA	25.7 ± 3.7	25.3 ± 3.1†	G x T	0.67
**Total Body Water (L)**	PRE	43.7 ± 13.9	45.4 ± 13.6†	Time	0.02
	POST	48.2 ± 13.9	51.6 ± 13.6†	Group	0.61
	PLA	45.9 ± 11.7	46.9 ± 11.2†	G x T	0.47
**Intracellular water (L)**	PRE	25.9 ± 8.6	27.5 ± 8.7†	Time	0.006
	POST	28.5 ± 8.3	31.4 ± 8.1†	Group	0.59
	PLA	26.9 ± 7.2	28.0 ± 7.1†	G x T	0.49
**Extracellular water (L)**	PRE	18.1 ± 5.6	17.8 ± 5.0	Time	0.77
	POST	19.7 ± 5.8	20.2 ± 5.6	Group	0.65
	PLA	18.9 ± 4.6	18.9 ± 4.4	G x T	0.46
**3CFIELD Fat Mass (kg)**	PRE	22.2 ± 4.9	20.3 ± 6.3†	Time	<0.001
	POST	22.2 ± 11.6	17.7 ± 10.3†	Group	0.57
	PLA	18.8 ± 7.9	16.6 ± 6.1†	G x T	0.33
**3CFIELD Fat-Free Mass (kg)**	PRE	58.8 ± 18.9	59.9 ± 18.3†	Time	0.04
	POST	65.1 ± 17.8	68.1 ± 17.7†	Group	0.60
	PLA	61.1 ± 16.1	62.9 ± 15.3†	G x T	0.11
**3CFIELD Body Fat (%)**	PRE	28.1 ± 4.3	25.7 ± 6.3†	Time	<0.001
	POST	24.8 ± 7.3	20.0 ± 7.6†	Group	0.18
	PLA	23.6 ± 7.4	21.2 ± 6.9†	G x T	0.38

† = Different (p<0.05) than respective baseline value using paired samples t-test. G x T = Interaction effect between Time and Group main effects. *p* = probability level of making Type I error.
