# Supplementary material for: Effects of creatine monohydrate timing on resistance training adaptations and body composition after 8 weeks in male and female collegiate athletes
Source: Front Sports Act Living. 2022 Nov 16;4:1033842. doi: 10.3389/fspor.2022.1033842 (PMC9708881; doi:10.3389/fspor.2022.1033842)
Supplement: Supplementary file 3 [file Table_3.pdf]

Supplementary Data Table 3. Performance Variables.

Variable	Group	Baseline (Week 0)	Post-Test (Week 8)		*p*
**Back Squat 1RM (kg)**	PRE	113.6 ± 58.7	122.7± 55.5†	Time	<0.01
	POST	141.9 ± 54.2	148.8 ± 58.9†	Group	0.53
	PLA	131.6 ± 55.6	140.7 ± 59.1†	G x T	0.87
**Back Squat Reps to Fatigue**	PRE	5.9 ± 2.7	6.4 ± 2.5	Time	0.98
	POST	7.7 ± 2.5	7.2 ± 2.7	Group	0.19
	PLA	8.0 ± 2.8	8.1 ± 4.9	G x T	0.82
**Back Squat Volume**	PRE	543.2 ± 418.2	612.9 ± 344.4	Time	0.45
	POST	888.2 ± 500.7	824.2 ± 472.2	Group	0.19
	PLA	831.8 ± 427.1	1012.6 ± 850.1	G x T	0.49
**Back Squat Strength / Mass Ratio**	PRE	1.5 ± 0.5	1.6 ± 0.5†	Time	<0.01
	POST	1.6 ± 0.5	1.7 ± 0.5†	Group	0.76
	PLA	1.6 ± 0.5	1.7 ± 0.6†	G x T	0.71
**Bench Press 1RM (kg)**	PRE	72.7 ± 39.7	75.4 ± 42.4†	Time	0.04
	POST	89.7 ± 47.4	92.4 ± 47.8†	Group	0.63
	PLA	79.3 ± 38.7	80.6 ± 39.2†	G x T	0.80
**Bench Press Reps to Fatigue**	PRE	7.8 ± 2.8	6.9 ± 2.2	Time	0.13
	POST	7.8 ± 3.6	5.7 ± 2.4	Group	0.79
	PLA	7·1 ± 2.6	7.5 ± 2.4	G x T	0.17
**Bench Press Volume**	PRE	424.2 ± 246.9	387.3 ± 218.9	Time	0.15
	POST	570.9 ± 417.5	427.7 ± 276.3	Group	0.69
	PLA	459.1 ± 278.5	471.1 ± 244.9	G x T	0.26
**Bench Press Strength / Mass Ratio**	PRE	0.9 ± 0.3	0.9 ± 0.4†	Time	<0.01
	POST	1.0 ± 0.4	1.0 ± 0.4†	Group	0.69
	PLA	0.9 ± 0.4	0.9 ± 0.4†	G x T	0.87
**IMTP Peak Power (Nm)**	PRE	464.4 ± 208.1	483.9 ± 226.0	Time	0.83
	POST	544.4 ± 174.3	526.0 ± 170.9	Group	0.66
	PLA	473.6 ± 173.7	464.9 ± 175.1	G x T	0.38

† = Different ($p<0.05$) than respective baseline value using paired samples t-test. G x T = Interaction effect between Time and Group main effects. *p* = probability level of making Type I error.
